# Multimodal and multidomain lesion network mapping enhances prediction of sensorimotor behavior in stroke patients

**DOI:** 10.1038/s41598-022-26945-x

**Published:** 2022-12-27

**Authors:** Antonio Jimenez-Marin, Nele De Bruyn, Jolien Gooijers, Alberto Llera, Sarah Meyer, Kaat Alaerts, Geert Verheyden, Stephan P. Swinnen, Jesus M. Cortes

**Affiliations:** 1 Computational Neuroimaging Group, Biocruces-Bizkaia Health Research Institute, Biocruces Bizkaia, Plaza de Cruces S/N, 48903 Barakaldo, Spain; 2 Biomedical Research Doctorate Program, University of the Basque Country (UPV/EHU), Leioa, Spain; 3 Department of Rehabilitation Sciences, KU Leuven, Leuven, Belgium; 4 Movement Control and Neuroplasticity Research Group, Department of Movement Sciences, KU Leuven, Leuven, Belgium; 5 LBI-KU Leuven Brain Institute, Leuven, Belgium; 6 Donders Institute for Brain, Cognition and Behaviour, Centre for Cognitive Neuroimaging, Nijmegen, The Netherlands; 7 Department of Cognitive Neuroscience, Radboud University Medical Centre, Nijmegen, The Netherlands; 8 LIS Data Solutions, Machine Learning Group, Santander, Spain; 9 Cell Biology and Histology Department, University of the Basque Country (UPV/EHU), Leioa, Spain; 10 IKERBASQUE, The Basque Foundation for Science, Bilbao, Spain

**Keywords:** Network models, Neural circuits

## Abstract

Beyond the characteristics of a brain lesion, such as its etiology, size or location, lesion network mapping (LNM) has shown that similar symptoms after a lesion reflects similar dis-connectivity patterns, thereby linking symptoms to brain networks. Here, we extend LNM by using a multimodal strategy, combining functional and structural networks from 1000 healthy participants in the Human Connectome Project. We apply multimodal LNM to a cohort of 54 stroke patients with the aim of predicting sensorimotor behavior, as assessed through a combination of motor and sensory tests. Results are two-fold. First, multimodal LNM reveals that the functional modality contributes more than the structural one in the prediction of sensorimotor behavior. Second, when looking at each modality individually, the performance of the structural networks strongly depended on whether sensorimotor performance was corrected for lesion size, thereby eliminating the effect that larger lesions generally produce more severe sensorimotor impairment. In contrast, functional networks provided similar performance regardless of whether or not the effect of lesion size was removed. Overall, these results support the extension of LNM to its multimodal form, highlighting the synergistic and additive nature of different types of network modalities, and their corresponding influence on behavioral performance after brain injury.

## Introduction

Mapping the behavioral impact of brain lesions is of vital importance in clinical practice. Regardless of the cause of the lesion, its size or precise location, evidence has accumulated in recent years in favor of the connectome hypothesis whereby the specific network(s) affected by the lesion can predict many of the patient’s responses through motor, non-motor, cognitive and behavioral domains^1–23^, leading to lesion-driven disconnectivity analyses^24^. A common computational framework was developed recently^2^, and successfully applied to several conditions and pathologies^3,4,6,7,9–18,22,25^. Due to the simplicity of this method to correlate behavioral outcomes with the extent of lesion-driven disconnection, the strategy was referred to as lesion network mapping (LNM). Here, we extend the classical LNM in to two strategic dimensions. Firstly, by proposing a multimodal strategy in which we introduce a combination of both structural and functional networks to predict behavior. Secondly, and motivated by previous work^5,20,21^, by assessing behavioral performance using a combination of several multidomain scores.

We applied our strategy to stroke, a highly disabling condition that typically produces multiple behavioral deficits. Even when stroke produces a focal damage, it is well known to affect remote areas, such as those regions directly connected to the lesion through long-range white matter tracts^26^ or connected regions indirectly by functional connectivity through the so-called common-neighbor interactions^27–29^. Significantly, the mapping of different behavioral deficits to imaging alterations localizes tightly within specific brain networks^5,21,30,31^. Indeed, the degree of network disruption was shown to be a good correlate of behavioral recovery from damage after stroke^32–34^, as also witnessed in longitudinal data^35–37^.

As stroke is a highly disabling condition, we assessed different aspects of the individual’s motor ability using two tests widely recognized as measures of motor performance: the Action Research Arm Test (ARAT) and the Fugl-Meyer assessment—upper extremity (FMA-UE) test^38,39^. In terms of everyday activities that involve different movement functions like grasping, grip and pinchforce, as well as gross movement, ARAT serves to measure upper limb dysfunction after stroke^38,40,41^. In addition, the FMA-UE test was used as a complementary assessment of motor dysfunction^42^. Somatosensory performance is also known to be highly impaired following stroke and it is generally associated with a deterioration in dexterity, manipulation abilities and bimanual hand coordination skills^43,44^. To assess somatosensory capacity, we used the Erasmus-modified Nottingham Sensory Assessment (Em-NSA)^45,46^ that evaluates tactile, proprioceptive and higher cortical somatosensation, along with the perceptual threshold of touch (PTT)^47^ that principally assesses tactile function and that has been used previously in neuroimaging studies^48^.

Our main hypothesis here was that by extending LNM using a multimodal strategy, similar to recent work^49^ combining information of lesion disconnectivity of functional and structural networks, it would facilitate a better understanding of the synergistic contributions of individual modalities to explain multi-domain sensory-motor outcomes in stroke patients. We also hypothesized that by applying multimodal LMN, the variance explained in the brain maps would be enhanced by achieving greater coverage of the variation in multi-domain sensory-motor behavior in stroke patients. For this purpose, we combined structure–function disconnectivity maps and employed a canonical correlation analysis (CCA) to link multi-domain behavior to different lesion connectivity maps.

## Results

Multimodal LNM was applied to a cohort of first-time stroke patients with sensory-motor impairments (N = 54). Behavior alterations were evaluated with a battery of sensorimotor tests. Demographic, clinical and sensorimotor scores are given in Table 1.

**Table 1:** Demographics, clinical characteristics and sensorimotor outcomes in stroke patients.

Variable, units	Mean	Sigma	Range
Age, years	68.78	13.98	28–92
Males/females	25/29	NA	NA
Time between stroke and assessment, days	25.61	20.32	4–64
Lesion size, cm^3^	45.71	58.95	0.30–255.94
ARAT	15.20	19.52	0–57
FMA-UE	26.22	20.61	0–59
Em-NSA	28.57	14.21	0–40
PTT	6.68	2.95	1.8–11

**Figure 1:**
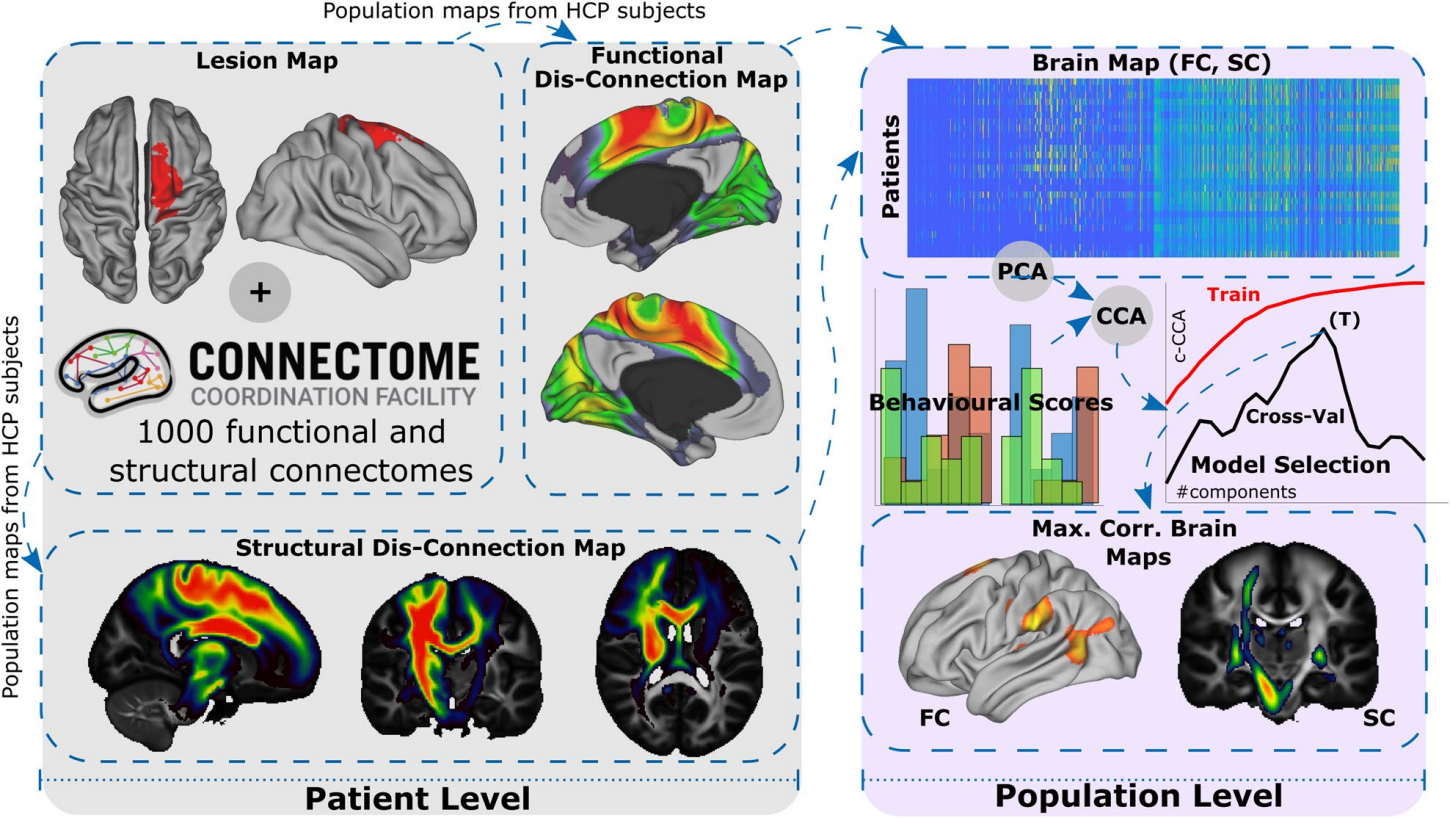
Pipeline for multimodal and multidomain lesion network mapping and its association to behavioral outcome after stroke through Canonical Correlation Analysis (CCA). At the patient level (gray shading), brain lesion masks are used as seed regions to calculate the functional correlation maps (applying seed-based correlation analysis and using the segmented lesion as the seed for each HCP subject) and the structural correlation maps (applying tractography from the segmented lesion to the rest of the brain for each HCP subject) from a group of healthy control participants from HCP (N = 1000). After averaging all the participants in the HCP dataset (see Methods for details), we obtained the functional disconnection maps for each patient, accounting for the functional impact of lesion disconnection, and likewise for the structural disconnection maps. At the population level (purple shading), a matrix with dimensions (# of stroke patients) x (# of voxels) per modality map (FC or SC) was built and reduced using a PCA, which returns a new matrix with (# of patients) × (# of principal components) dimensions, the PCA components considered here as the brain map features. The association between the features of the SC and FC, and the behavioral scores was obtained by applying a CCA. As the number of features increases, the correlation between features and behavior (represented here as c-CCA) increases up to values close to 1 (red curve, Train), dealing with overfitting. Cross-validation techniques can overcome this problem (for details see Methods). For the maximum CCA correlation value in the cross-val curve (black), represented by T, we built brain maps of those components producing maximum performance. The maps can be obtained in a single modality, here shown for FC or SC, or as a combination of them (not shown here but implemented in this study).

After projecting all the patient’s lesions onto the same template (MNI152, 2 mm^3^), we obtained the functional and structural disconnection maps for each patient following the pipeline detailed in Fig. 1. This pipeline also made use of functional and structural imaging data from healthy HCP participants (N = 1000). Thus, our method analyzed the impact that the patient's lesion had on the disconnection of specific networks that exist in healthy brains. At the population level (N = 54 stroke patients), we concatenated the different patient’s disconnection maps into a final matrix and applied a PCA to get the principal components that were then used as independent variables for the CCA.

**Figure 2:**
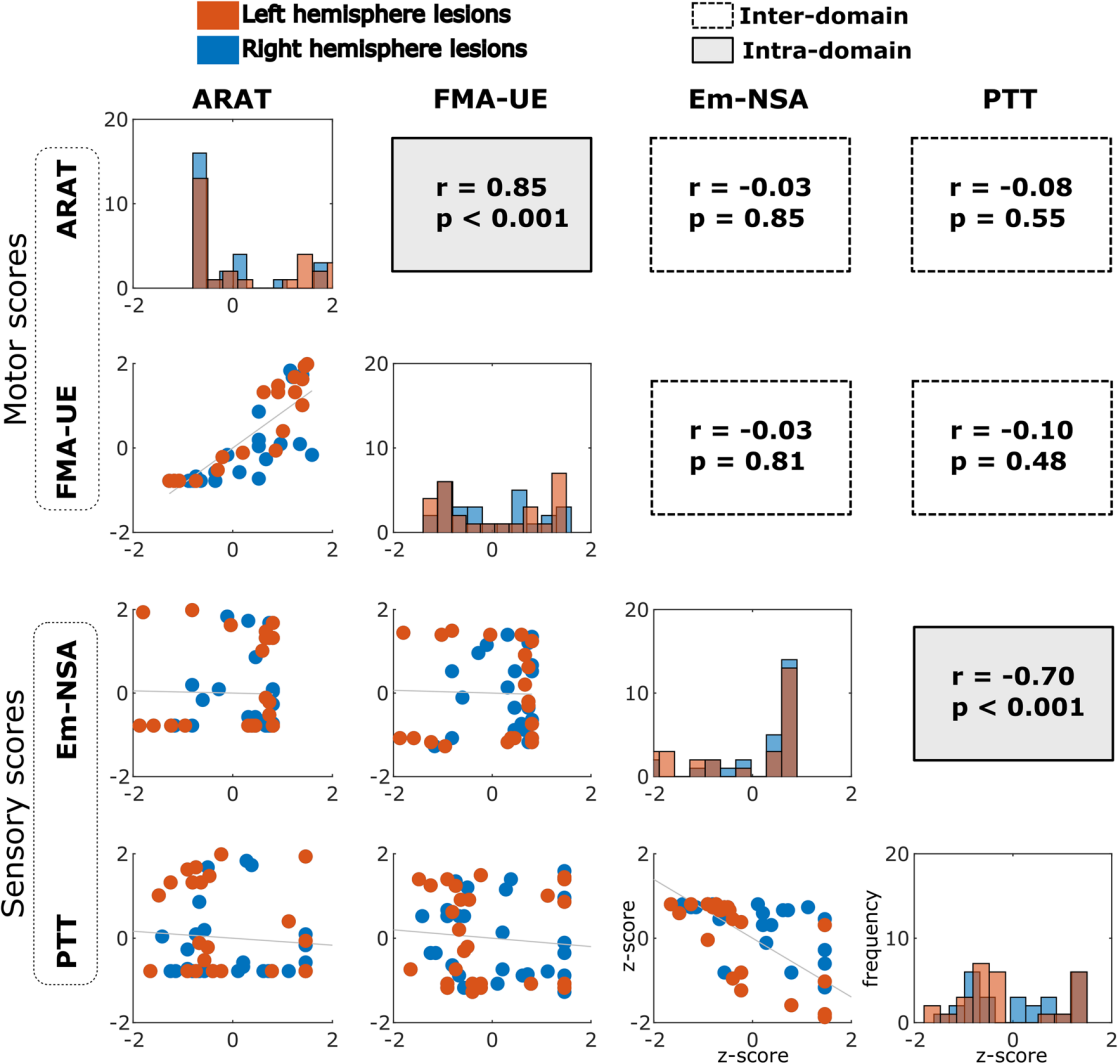
Distribution of behavioral—motor and sensory—scores. The principal diagonal panels represent the histogram values for each of the four behavioral scores, two being motor scores (ARAT and FMA-UE) and two somatosensory scores (Em-NSA and PTT). Off-diagonal panels (below the diagonal) show scatter plots between pairs of scores. We also provided Pearson correlation values (r) and associated p-values above the diagonal in the off-diagonal panels. The red and blue colors represent scores from patients having lesions in the left and right hemispheres, respectively. Behavioral outcome differences between patients with left and right hemisphere lesions were not significant (ARAT χ^2^ = 0.85, p = 0.36; FMA-UE χ^2^ = 0.003, p = 0.96; Em-NSA χ^2^ = 0.69, p = 0.41; PTT χ^2^ = 0.44, p = 0.51). Because these reasons, the left and right lesion datasets were pooled into a single cohort in this study. All the scores are represented here as Z-scores.

The dependent variables for the CCA were the behavioral sensorimotor scores (Fig. 2). The two ARAT and FMA-UE motor scores were correlated to each other (*r* = 0.85, *p* < 0.001), as were the sensory Em-NSA and PTT variables (*r* = − 0.7, *p* < 0.001). However, the correlations between motor and sensory variables were not significant (ARAT vs. Em-NSA *p* = 0.85; ARAT vs. PTT *p* = 0.55; FMA-UE vs. Em-NSA, *p* = 0.81; FMA-UE vs. PTT, *p* = 0.48), suggesting linear independence between the two motor and somatosensory domains. In addition, the differences in the scores between patients with left hemisphere lesions as opposed to those with right hemisphere lesions were not statistically significant (ARAT *χ*^2^ = 0.85, *p* = 0.36; FMA-UE *χ*^2^ = 0.003, *p* = 0.96; Em-NSA *χ*^2^ = 0.69, *p* = 0.41; PTT *χ*^*2*^ = 0.44, *p* = 0.51). Indeed, there was a high similarity between the lesion brain maps in the left hemisphere and those of the right (*r* = 0.71, *p* < 0.001: Fig. S1). Both factors (the non-significant differences between behavioral scores in patients with left or right hemisphere lesions, and the high similarity between the two lesion spatial maps) justified merging patients with left and right hemisphere lesions into a single cohort, thereby increasing the statistical power for our CCA analysis.

We next analyzed the statistical relationship between behavioral scores and confounding factors of age, time between stroke and the behavioral assessment (TBSAA), and lesion size (Figs. S2 and S3). The two latter variables showed significant correlations with the somatosensory outcome assessed by Em-NSA (*r* = 0.32, *p* = 0.02 and *r* = − 0.45, *p* < 0.001, respectively), while lesion size was correlated with the PTT score (*r* = 0.42, *p* = 0.002). The three co-variables age, TBSAA and lesion size where regressed out from the sensorimotor scores for the following analyses.

**Figure 3:**
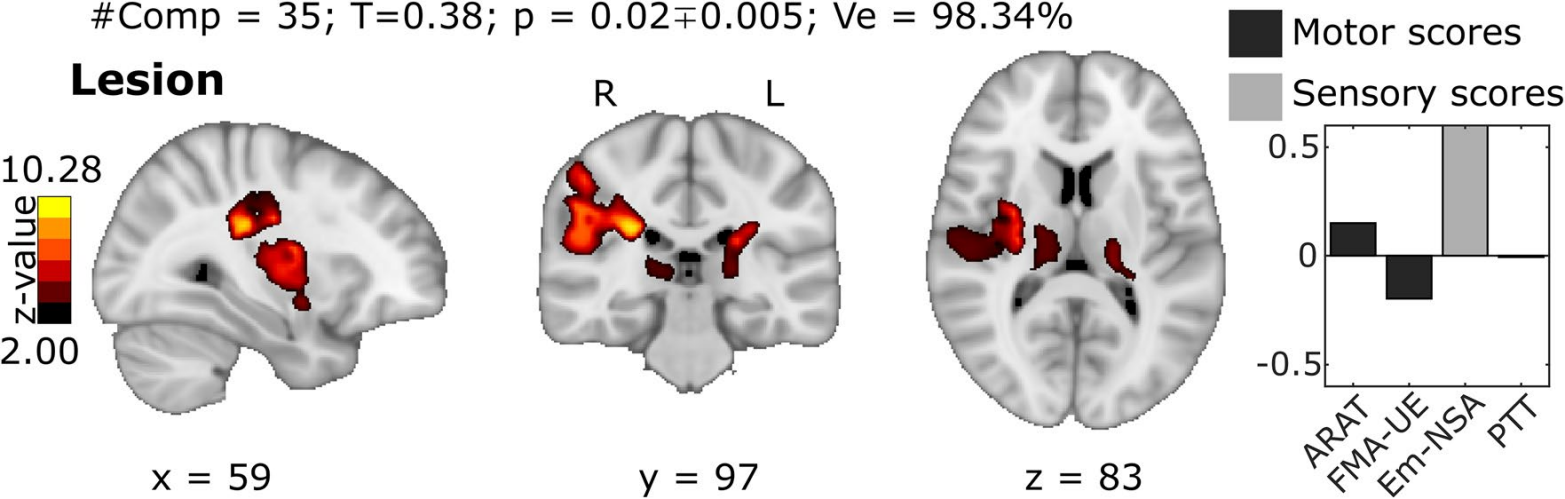
Brain maps with maximal behavioral association from Lesion Symptom Mapping and CCA. Final map corresponding to the CCA solution and providing the maximum correlation between the X variables (the PCA components from lesions maps) and the Y variables (a combination of several behavioral scores represented in Fig. 2). Together with the map, we provide the number of PCA components used (#Comp), the maximum correlation value (T), p-value (p) with the error intervals, and the amount of variance explained (Ve). For visualization, the map was threshold to $Z>2$, but a complete map without thersholding is given in Fig. S5. In the left panel we represent the behavioral weights corresponding to the maximum behavioral-association solution.

**Figure 4:**
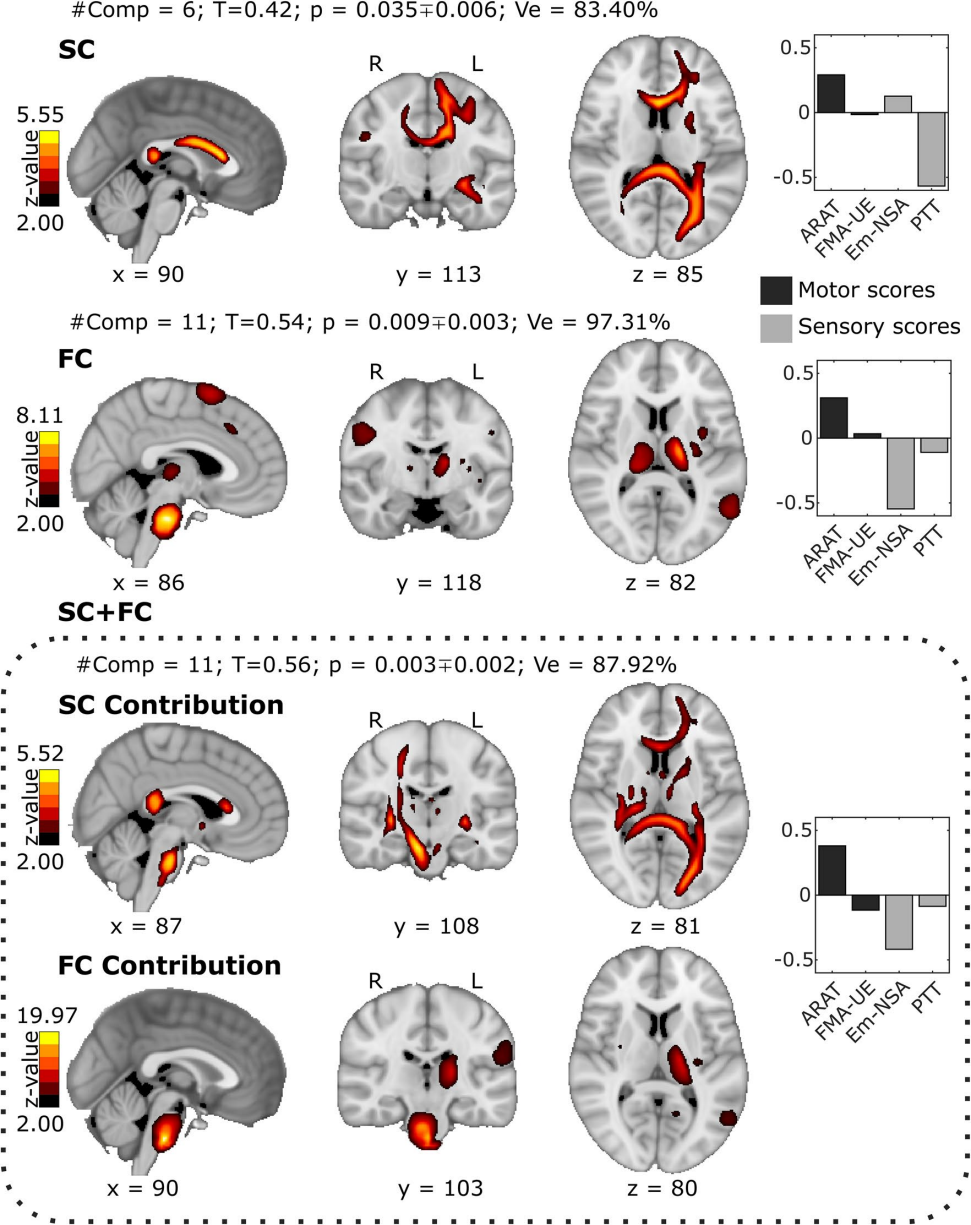
Brain maps with a maximal behavioral association from the unimodal and multimodal Lesion Network Mapping and CCA. Final maps of the CCA solution that provide the maximum correlation between the X variables (the PCA components from each modality) and the Y variables (a combination of several behavioral scores represented in Fig. 2). From top to bottom, SC brain maps (accounting for SC disconnectivity), FC brain maps, and SC + FC multimodal maps (box with dashed line). Moreover, the bottom panel also shows the individual SC and FC contributions to the maximum performance achieved by the multimodal SC + FC strategy. Together with the maximum behavioral-association maps, in all cases we provide the number of PCA components used (#Comp), the maximum correlation value (T), p-value (p) with the error intervals, and the amount of variance explained (Ve). For visualization, all maps were threshold to $Z>2$, but complete maps with no thersholding are given in Fig. S5. In the left panel of each row we represent the behavioral weights corresponding to the maximum behavioral-association solution.

Before studying the extent to which the disconnection caused by the lesion explained the sensorimotor outcome, we applied lesion symptom mapping (LSM), in which only the location and size of the lesion are taken into account (but not its connectivity) to predict behavior. In particular, we follow LSM similar to^50^ but extending it to multi-domain sensorimotor behavior, for the purpose of comparing the performance of LSM with that of LNM (using the network disconnection paradigm). In all cases studied, including LSM and LNM (unimodal or multimodal), we followed a similar computational pipeline consisting of an iterative CCA. In particular, after sequentially increasing the number of PCA components, the best cross-validation curve model (black curve in Fig. S4) was chosen, selecting the number of components (#Comp) that yields the maximum significant correlation (T) through CCA. The resulting brain maps (Z > 2) are illustrated in Fig. 3 for LSM and Fig. 4 for LNM, presenting the results for unimodal (FC or SC) versus multimodal (FC + SC). The results with Z without threshold are also shown in Fig. S5. In addition, the amount of explained variance (Ve) in #Comp for each case is also shown (Figs. 3 and 4).

LSM maps corresponding to the highest behavioral association provided brain regions in both grey matter (Table S1) and white matter (Table S2). The GM regions were composed majorly of bilateral supramarginal gyrus, bilateral insula, bilateral thalamus, right postcentral, and right precentral. The WM regions coincided majorly with bilateral arcuate fasciculus, bilateral superior thalamic radiation, and bilateral corticospinal tract.

Highest behavioral association LNM maps obtained from SC lesion-disconnectivity (Tables S3–S4) revealed major tracts participating in unimodal and multimodal analyses were the forceps major, left frontal aslant tract, left anterior thalamic radiation, bilateral superior longitudinal fasciculus and bilateral optic radiation. Importantly, some tracts in the brain only appeared to participate during multimodal association, specifically, the right corticospinal tract and the middle cerebellar peduncle. Next, when looking at the overlap between SC maps and major sensorimotor-tracts represented in the SMATT atlas (a more specific sensorimotor atlas, cf. Tables S5–S6), unimodal SC maps included the left dorsal and ventral premotor cortices, right pre-supplementary motor and right primary somatosensory cortex. By contrast, SC multimodal maps included the right primary motor area, right primary somatosensory cortex and left ventral premotor cortex. When comparing the significant association (Z > 2) between the two hemispheres (L and R), we found significant differences in the region of significant association measured by the number of voxels between unimodal analysis N = 12,203 (L) and N = 2589 (R) and multimodal N = 7450 (L) and N = 5606 (R). Hence, the proportion of left vs. right voxels was significantly lower in the multimodal as compared to the unimodal (*χ*^2^ = 2160.04, *p* < 0.001).

Highest behavioral association LNM maps obtained from FC lesion-disconnectivity revealed that brain regions that participated in both unimodal and multimodal associations were the brainstem (specifically the pons), left supramarginal gyrus (the part overlapping with the secondary somatosensory cortex), left thalamus, bilateral superior frontal cortex (overlapping with the premotor cortex and supplementary motor area), left inferior parietal and right precentral cortex (overlapping with the primary motor cortex and primary sensory cortex). For a complete description of all anatomical areas with significant association see Tables S7–S8. More specifically, when looking at the overlap between FC maps and the major Resting State Networks (RSNs: Tables S9–S10), the unimodal FC maps overlapped with the dorsal attention and limbic networks, while a major participation for ventral attention and sensorimotor networks was evident in the FC multimodal analysis. Similar to what happened for SC, when the region of significant association in the two hemispheres was compared, we found N = 4023 (L) and N = 3269 (R) significant voxels in the unimodal analysis, and N = 3369 (L) and N = 1958 (R) for multimodal, confirming an opposite trend as in SC, namely, we have a lower proportion of left vs. right voxels in the unimodal as compared to the multimodal (*χ*^2^ = 82.70, *p* < 0.001).

When looking to the behavioral weights corresponding to the *best* model solution (Figs. 3 and 4), we found LSM and unimodal LNM analyses, but not multimodal LNM, to have an imbalance in the optimal weights, with greater weights assigned to the somatosensory domain than to the motor domain. By contrast, the LNM multimodal solution provided a more balanced state between motor and somatosensory contributions. Moreover, the individual contributions of SC and FC within the multimodal *best* solution had different strengths, as witnessed by the median value of the weight distribution for the SC (0.08) and FC (0.12), and indicating a major participation of FC as opposed to SC in the multimodal solution. Furthermore, when comparing the performance of the behavioral association between the unimodal and multimodal analyses, FC outperformed SC at approximately 30% higher values of maximum correlation and at 16% higher values of explained variance.

In relation to performance, both the unimodal LNM SC or unimodal LNM case and the multimodal LNM SC + FC case worked better than LSM (LSM T = 0.38 vs. LNM unimodal SC T = 0.42, LNM unimodal FC T = 0.54, LNM multimodal SC + FC T = 0.56). Therefore, network data improved prediction beyond lesion location. Moreover, the maximum performance corresponded to multimodal LNM. Finally, in an attempt to address which modality was more dependent to the elimination of the effect of lesion size, we repeated a similar strategy as before but without eliminating such effect (Fig. S6 for LSM and Fig. S7 for LNM). In this case, we found that LSM and unimodal LNM SC, both had greater variation in performance when eliminating the effect of lesion size (LSM eliminating T = 0.38 vs not eliminating T = 0.61; unimodal LNM SC eliminating SC = 0.42 vs not eliminating T = 0.63). On the other hand, the unimodal FC LNM practically maintained the same performance when eliminating or not eliminating the effect of the size of the lesion (eliminating T = 0.54 vs not eliminating T = 0.56). These results indicated that both SC disconnection maps and lesion maps are more dependent to lesion characteristics than FC maps. Finally, we found that in any of the four cases studied (LSM, unimodal LNM SC, unimodal LNM FC and multimodal LNM SC + FC), and independently on eliminating or not the effect of the size of the lesion, the best performance occurred for the multimodal LNM.

## Discussion

Lesion network mapping (LNM) is a novel technique based on brain connectivity that it is conceptually straightforward and has shown impressive performance in the study of brain-behavior interactions^51^. Critically, LNM makes use of only patient's lesion data and reconstructs which brain regions in a healthy population of young adults (N = 1000) are putatively disconnected by the patients’ lesion. By this simple association or correspondence, LNM has been capable of explaining the presence or absence of a similar symptom within a clinical cohort, depending on whether or not the patients' lesions are connected to a common network. Importantly, a recent review has shown that while LSM was not sufficient to explain motor, non-motor, sensory and behavioral changes after brain damage, LNM successfully explained a total of 40 different changes^52^, relating symptoms to lesions connected to a common network, and where more often than not, the location of the lesion alone (the gold standard in clinical practice) was not able to explain these symptoms.

In relation to this, recent data have linked LNM with improvement in behavior^53^, linking networks resulting from LNM as candidates to explain behavioral changes with better outcome after deep brain stimulation (DBS) in patients with tics. In particular, patients with electrode locations connected to LNM had a better outcome after DBS, regardless of whether or not the stimulation was targeted to the thalamus or globus pallidus, thus reinforcing the clinical meaning of LNM independently to which brain structure is connected with.

In other words, the work in^53^ showed that the predicted LNM has physiological significance, because patients who responded well to DBS had electrodes connected to LNM.

In this way, LNM has shown to stratify lesion-triggered symptoms through differentiated network mapping, offering two direct advantages over other *patient-specific* brain connectivity approaches (using the same terminology as in^20^, they refer to the direct method when patient connectivity data is used, in contrast to an indirect method, where only normative connectivity data is used in combination with patient lesion data). The first advantage of LMM is technical, as the reconstruction of networks in lesioned brains remains a challenge^54^, and this step is not necessary for LNM. The second advantage of LNM is practical, as LNM can be performed simply by acquiring a few FLAIR or T2 slices around the lesioned area, rather than having to acquire a full multimodal MRI protocol. Our contribution here further extends the classical LNM in two critical directions. First, by performing fusion of FC and SC networks similar to recent work^49^, we assess the multimodal impact of a stroke lesion in predicting behavior. The incorporation of other modalities within the multimodal strategy would be straightforward simply by spatial concatenation of the connectivity matrix of each individual. Second, we study the association between multimodal networks and multidomain sensorimotor behaviour, since the dysfunctionality caused by stroke affects different behavioral domains. Therefore, a systemic characterization of patients requires the use of methods that can deal with this behavioral complexity. In the present work, these two aspects of multimodal and multi-domain are combined in an original manner using CCA, a successful paradigm to map brain images onto behavior^55–58^.

Our multimodal LNM results highlight several improvements when compared to classical LSM and the unimodal LNM approach. First, multimodal LNM reveals that functional maps contribute more strongly than structural maps to the optimal prediction of sensorimotor behavior. FC provided highest explained variance than SC, which might reflect the fact that it is easier to adjust FC than SC after the brain injury. In relation to the number of behavioral domains covered by the best solution to link brain maps and behavior, we found that while LSM, unimodal LNM SC and unimodal LNM FC better captured the sensory domain, the multimodal LNM provided a more balanced sensorimotor representation, supporting the notion that multidomain behavior is better represented on multimodal circuits. Finally, when looking at the shared variance of the multimodal analysis—across functional and structural data at the patient level—, we unveiled the participation of some regions and networks that did not appear in the unimodal analyses, such as the corticospinal tract (CST) and the primary motor area (M1), and the middle cerebellar peduncle (MCP), the two former CST and M1 well-known critical structures for motor function. In addition, MCP mediates the communication between the cerebellum and the prefrontal cortex in the coordination and planning of motor tasks^59^. Moreover, the networks emerging from the multimodal analysis were the ventral attention network (VAN) and sensorimotor network, the former known to be relevant for stimulus driven attention, such as when somatosensory input is being processed^60^.

Another issue to highlight is what happened when we removed the effect of lesion size from our analyses, as the disconnectivity amount was expected to be higher in bigger lesions. Unimodal LNM SC and LSM were highly dependent on this step and their performance deteriorated dramatically after lesion size was removed, as witnessed here by the amount of variance explained and the maximum correlation with behavior achieved. By contrast, FC provided similar performance irrespective of any correction for lesion size, suggesting that the FC is less localized (or more redundant) when processing sensorimotor tasks relative to the structural maps.

An interesting observation from our data was that lesion size was significantly correlated with the two sensory tests (Em-NSA and PTT) but not with either of the two motor tests (ARAT and FMA-UE), in agreement with previous work^61^. These results show that the motor outcome assessed in this study was apparently processed in more localized brain regions that, once affected, would provoke a worse outcome regardless of the lesion size. Conversely, because lesion size was correlated with sensory outcomes, it suggests that the sensory consequences of the lesion are distributed more broadly across circuits encompassing different brain areas (visual—parietal—frontal), in agreement with previous results showing a stronger interaction between brain areas for sensory processing than for motor tasks^62^. This phenomenon might also be related to the amount of cognitive intervention required. While motor outcomes can be processed in a more straightforward manner, the somatosensory outcomes require the recruitment of more resources involved in cognitive control, including those required for attention deployment, although this possibility clearly requires further clarification.

Recent work acknowledged certain limitations to LNM^63,64^. For example, when dealing with large lesions containing both white and grey matter, using the segmented whole lesion as the only seed to perform LNM can introduce relevant methodological biases due to the differences in the BOLD signal between voxels belonging to gray or white matter. To address FC, here we adopted a more fine-grained approach by averaging the signals within the gray matter. In relation to our study, a limitation is that our clinical population is highly heterogeneous with regards to TBSAA, in this sample raging within the acute to sub-acute recovery epochs (4–64 days). Although we have used TBSAA as a covariate for all of our analyses, and therefore we were correcting for this effect, we cannot in any manner assess chronic sensorimotor outcome (e.g., > 12 months), although our methodology is perfectly valid to be applied to such data, which should be answered in future studies. A second limitation is that the stroke patients studied were recruited paying attention to whether they had sensory impairment and independently whether they suffered any effect in motor performance, which might introduce some bias with respect to other studies in which patients with greater motor dysfunction were recruited.

## Conclusion

By applying a multimodal and multidomain LNM approach, we have predicted sensorimotor behavior, showing evidence about the synergistic and additive role of different types of brain networks on patients after brain injury, affecting their outcome and thereby making the whole more than the sum of its parts. Moreover, when a patient's behavior is assessed across multiple domains of cognitive, sensory and motor function, our methodological approach, combining structural and functional maps, appears to be the most suitable and clinically relevant for assessing such multidomain outcomes.

## Methods

### Participants

In this study we included two cohorts of stroke patients previously assessed elsewhere^65,66^. The first cohort consisted of 25 patients who developed upper limb sensorimotor impairments after stroke, and they were recruited from the University Hospital Leuven and the University Hospital St-Luc Brussels. The second cohort consisted of 29 stroke patients recruited at four different centers: UZ Leuven (Pellenberg), Jessa hospitals (Herk-de-Stad), Heilig Hart Hospital (Leuven) and RevArte (Antwerp). The two cohorts were combined into one, improving the statistical power of the analyses. Thus, in total we analyzed 54 first-stroke patients (25 males) with a mean age of 68.78 (σ = 13.98, range 28–92 years), and a mean time interval between stroke and behavioral assessment of 25.61 days (σ = 20.32, range 4–64). The lesions were distributed across the left and right hemispheres (27 lesions in each hemisphere) and the size of the lesions varied from 0.30 to 255.94 cm^3^. Due to the large variation in time between stroke and behavioral assessment (TBSAA), and in the participant’s age and lesion size, these three covariables were regressed out for further analyses. The behavior of both cohorts of patients was assessed at the hospital using a dedicated procedure not included in the daily clinical routine. Full details of the demographic and clinical characteristics of the participants in this study are given in Table 1.

### Ethics declarations

The study was approved by the Ethical Committee of UZ/KU Leuven (codes S60278 and S54601) and all the participants provided their signed informed consent before enrolling on the study. All methods were performed following all approved recommendations by the ethical committee.

### Image acquisition

T1 anatomical MRIs were acquired from all the stroke patients (N = 54) using a Philips 3 T Achieva scanner equipped with a 32-channel head coil and applying the following parameters: 182 coronal slices covering the whole brain, repetition time (TR) = 9.6 ms, echo time (TE) = 4.6 ms, field of view (FOV) = 250 × 250 mm^2^, slice thickness = 1.2 mm and no interslice gap. FLAIR images were acquired with the following parameters: 321 transverse slices covering the whole brain, TR = 4800 ms, TE = 351 ms, inversion time = 1650 ms, FOV = 250 × 250 mm^2^, slice thickness = 1.12 mm and interslice gap = 0.56 mm. For the first cohort, we only acquired the FLAIR sequence. These images were used only for lesion segmentation.

We also analyzed images from healthy subjects (N = 1000, ages ranging from 22 to 35 years old) obtained from the Human Connectome Project (HCP, WU-Minn Consortium, Principal Investigators David Van Essen and Kamil Ugurbil: 1U54MH091657) funded by the 16 NIH Institutes and Centers that support the NIH Blueprint for Neuroscience Research, and by the McDonnell Center for Systems Neuroscience at Washington University. For each HCP subject, MRI acquisition was performed using a 3 T Siemens Connectome Skyra with a 100 mT/m and 32-channel receive coils. The acquisitions used for network disconnectivity analyses were: (1) a high-resolution anatomical T1-weighted 3D MPRAGE sequence with the parameters TR = 2400 ms, TE = 2.14 ms, Flip angle = 8 deg, FOV = 224 $\times$ 224 mm^2^, Voxel size = 0.7 mm isotropic, Acquisition time = 7 min and 40 s; (2) functional data at rest to obtain the blood-oxygenation-level-dependent (BOLD) signals using a gradient-echo EPI sequence with the parameters TR = 720 ms, TE = 33.1 ms, Flip angle = 52 deg, FOV = 208 $\times$ 180 mm^2^, Matrix = 104 $\times$ 90, 72 slices per volume, a total number of 1200 volumes, Voxel size = 2 mm isotropic, Acquisition time = 14 min and 33 s; (3) diffusion weighted data with a Spin-echo EPI sequence and the parameters TR = 5520 ms, TE = 89.5 ms, Flip angle = 78 deg, FOV = 210 $\times$ 180 mm^2^, Matrix = 168 $\times$ 144, 111 slices per volume, Voxel size = 1.25 mm isotropic, 90 diffusion weighting directions and six unweighted (b = 0) acquisitions, three shells of b = 1000, 2000 and 3000 s/mm^2^, Acquisition time 9 min 50 s. For further details on the acquisition parameters of the HCP participants see the documentation available at https://www.humanconnectome.org/.

### Image processing

#### Lesion segmentation

Lesion segmentation was based on both T1 and FLAIR images, and it was performed semi-automatically using the *clusterize* toolbox^67^ implemented in SPM12 and running in MATLAB R2019b, followed by manual inspection and correction in MRIcron by experienced researchers. After segmentation, the lesion masks were non-linearly co-registered to the MNI152 template with the dimensions 2 × 2× 2 mm^3^. To enhance this co-registration we used the T1 sequence (or FLAIR in the first cohort), filling the lesioned area with healthy tissue from the contralateral hemisphere.

#### Functional images from healthy HCP participants

Resting state functional MRIs from HCP healthy controls (N = 1000) were used to generate functional connectivity maps of lesions. First, the images were corrected for EPI gradient distortions and normalized to the MNI152 standard template with a voxel size equal to 2 × 2 × 2 mm^3^ using the HCP *fMRIVolume* and *fMRISurface* pipelines. After image normalization, we removed all nuisances with a procedure that mixes a volume-censoring strategy and a movement-related time-course regression, together with physiological signal regression. To do so, volumes were marked as censored when the frame-wise displacements (FDs) were greater than 0.2 or the derivative of the root-mean-squared variance was greater than 0.75%, following previous recommendations^68–70^. Moreover, the volume prior to, and the two following the censored one, were also marked as censored. The entire time series was then split into segments of 5 volumes in length, to finally remove all segments containing at least one contaminated volume, as well as the first segment. The 1000 subjects selected were those with the least contaminated volumes. Subsequently, any nuisances were removed while simultaneously applying a bandpass filter between 0.01 and 0.08 Hz. Nuisance signals were the first five principal components of the CSF and white matter signals, the linear and quadratic trends, and the 24-parameter movement-related time-series. Finally, each filtered image was spatially smoothed with a Gaussian kernel of 6 mm FWHM.

After image preprocessing and to speed up computation, functional disconnection maps were obtained using only the first six minutes of the preprocessed 4D image. To do so, the lesion mask for each stroke patient was used as the seed for the analysis of seed-based connectivity (SBC), applied separately to each HCP subject. In such a way, the time-series of the BOLD signals were obtained from HCP data, while the stroke patients provided the seed for SBC. The Pearson correlation values, ‘$r$‘, between the seed-time series (obtained by averaging all the voxel-time series within a given lesion) and all other voxel-time series in the brain, were Fisher-transformed by applying the inverse hyperbolic tangent of $r,$ i.e., $z=artanh(r).$ Therefore, for each HCP subject and stroke patient we obtained a 3D brain map of z-values. The final functional disconnection map per patient was obtained after one-sample T-test statistics were applied to the 1000 HCP different maps.

#### Diffusion weighted images from healthy HCP participants

Inspired by the strategy to obtain functional disconnection maps, we applied SBC to the diffusion data in order to obtain structural disconnection maps. We first made use of the *bedpost*^71^ results obtained after applying the HCP pipeline to each subject. The Camino software (http://camino.cs.ucl.ac.uk/) was then used to obtain a deterministic tractography, with fiber assignment using a continuous tracking algorithm^72^ and all voxels within a given lesion as seeds for whole-brain fiber generation, employing a maximum curvature of 60° and a fractional anisotropy threshold of 0.15. The voxel-level fiber-counting maps were then binarized for each HCP subject, defining the extent to which a given voxel in the brain is connected to any voxel within the lesion. Finally, we obtained the final structural disconnection map by averaging across all HCP participants, one for each of the stroke patients in our cohort. Therefore, a given voxel in the final map had a value of 1 when the stroke patient's lesion was connected to that voxel in all HCP participants and conversely, 0 if it was not connected in any HCP participant.

### Sensorimotor assessment as a behavioral outcome in stroke patients

Somatosensory performance was evaluated using the Em-NSA^45^ to assess exteroception, proprioception and higher cortical functions. The Em-NSA evaluates five distinct somatosensory modalities^73^ including light touch, pressure, pinprick, sharp-blunt discrimination and proprioception. Light touch was tested with cotton wool, pressure with an index finger pinprick with a toothpick, and sharp-blunt discrimination by alternating a toothpick prick of the index finger with that at the following contact points: fingers, hand, forearm, and upper arm. Proprioception was assessed during passive movements of the different upper limb joints. Each point of contact was assessed 3 times and graded on an ordinal scale as: 0, patient fails to detect any sensation on all 3 occasions; 1, patient identifies test sensation, but not on all 3 occasions; or 2, patient correctly identifies the test sensation on all 3 occasions. In total, the scores for each modality ranged from 0 (complete somatosensory impairment) to 8 (no somatosensory impairment). The total score for the Em-NSA (including all modalities together) ranges from 0 to 40, with a higher score representing better upper-limb somatosensory performance and a score below 36 indicating a degree of somatosensory impairment^40^. A second test that was used for somatosensory performance was the PTT^47^, which assesses gentle touch perception by applying transcutaneous electrical nerve stimulation (TENS) with a CEFAR Primo Pro apparatus (Cefar medical AB, Sweden). The scores reflect the mA applied to detect stimulation on the affected tip of the index finger and thus, higher PTT scores indicate more somatosensory impairment, with maximal stimulation being set at 10 mA to prevent burning. If a patient was unable to feel any sensation at the maximum stimulation level they were awarded a score of 11.

Motor performance was assessed with the ARAT to evaluate upper limb activity^41^, a 19-item test divided into 4 categories: grasp, grip, pinch, and gross arm movement. Each category is rated with an integer value ranging from 3 (patient performs test with normal motor pattern) to 0 (patient cannot perform any part of the test). Therefore, the maximum score after the ARAT test is 57, indicating normal motor performance of the arm. Motor performance was also assessed with the FMA-UE^42^ test, addressing motor function of the upper extremity as a whole (including shoulder, elbow, wrist and hand movements), from reflex activity to voluntary activation^73^. The total FMA-UE score ranges between 0 and 66, with a higher score representing better upper-limb motor function.

In any case, for each participant the assessment was performed in their affected limbs.

### Association between behavioral outcomes and the disconnectivity maps

#### Sensory-motor data pre-processing

Because there were some missing values in the behavioral scores from stroke patients, we applied the following procedure to this data. Rather than penalizing the sample size by eliminating these patients, we generated the missing values using an iterative imputer algorithm with extra tree regressors, seen to be highly effective in generating missing data^74,75^. The total number of missing values was zero for the ARAT test, 3 for the FMA-UE test, 2 for the Em-NSA and 2 for the PTT.

#### Canonical correlation analysis (CCA)

First, we merged the patients’ maps from lesions in the left and right hemisphere into a single cohort, thereby increasing the statistical power for our CCA analysis. Next, by identifying the set of voxels in each map with non-zero values from each subject and modality, we added all these voxels into a single mask for the entire population, and allowing us to obtain matrices with dimensions (# of stroke patients) × (# of voxels within the modality mask). The principal components (PC) of these matrices were used as the input of the CCA. In particular, we applied an iterative CCA to establish associations between imaging and behavioral outcomes. For this, the residuals of the behavioral variables were used as the dependent variables (after regressing out lesion size, patient age, and TBSAA) and the PCs as the independent variables, starting with one component as the X variable and ending with a matrix including all the PCs. When the number of components in X increased, the CCA strategy led to overfitting, making the extent of the correlation achieved meaningless. To overcome this limitation, we performed a predictive CCA approach using leave-one-out cross-validation (LOOCV). Thus, to obtain the CCA predicting correlation value, for every step (subject) we adjusted the CCA with *all-except-one* participants and predicted the canonical scores of the remaining participant with the *learned model*. Statistical significance was assessed by surrogate generation of 1000 random permutations of the X variables (we also applied 5000 and 10,000 surrogates and significance did not change), and the p-value estimated by counting the number of instances where the surrogate correlations were greater than those produced by CCA divided by the total number of permutations^76^, which in our case was sufficient for controlling all false positives in the null-distribution within our p-value *granularity*, determined by 1/#permutations which is the minimum achievable p-value. We also estimated p-value errors by calculating $\sqrt{p*(1-p)/\#perm}$^77^. Finally, we selected the *best* model as that for which the PCA order achieved the maximum correlation *T* using the predictive LOOCV-CCA introduced.

This procedure was performed for SC and FC separately (unimodal analyses), and combined through spatial concatenation of standarized SC and FC matrices (used for the multimodal analysis). This was followed by a PCA and the *mixed* components obtained were used for CCA, the same as for the unimodal cases. In the multimodal approach, to control for the variability and the differences in the range values between modalities, we first transformed the SC to logarithmic values, and then standardized both the SC and FC matrices before concatenation.

#### Brain maps corresponding to *best* CCA solutions

Using the weights of the *best* model and their coefficients, we projected this solution back onto the brain space. The final maps were obtained by transforming the values to z-scores and representing only the $Z>2$ values (the maps with no thresholding are also included in Fig. S5 for comparison purposes). For the multimodal strategy, we first back-projected the best solution, and then we split the different coefficients identifying the separate SC and FC contributions.

#### Neurobiological description of brain maps

Brain maps were described using four different atlases. The first one was XTRACT^78^, composed of 42 different white matter tracts, including: 10 association tracts (L/R hemispheres), 4 commissural tracts, 4 limbic tracts (L/R hemispheres), and 5 projection tracts (L/R hemispheres). The second atlas was Desikan-Killiany^79^ with 88 regions, 8 of them subcortical (L/R hemisphere) and 36 cortical (L/R hemispheres). To this atlas, we added the brainstem as an additional region. The third atlas was SMATT^80^ with 60 regions in total, 30 sensorimotor tracts (L/R hemispheres). Finally, the fourth atlas was an overlay of several partitions proposed by Yeo and collaborators, including the cortex^81^, cerebellum^82^, striatum^83^ and thalamus (to the best of our knowledge released by the author but not yet published in any citable reference).

### Statistical analyses

Unless otherwise specified, group comparisons of different metrics used in this study (e.g., scores in patients with a lesion in the right hemisphere compared to those in the left hemisphere) were performed with two-sample Kruskal–Wallis non-parametric tests, reported as χ^2^ stat. Statistical dependencies between behavioral scores were assessed through Pearson correlation analysis. Spatial similarity between brain maps was assessed using Pearson spatial correlation.

## Supplementary Information

Supplementary Information.

## Data Availability

MRI anatomical, functional and diffusion images from the 1000 healthy subjects are available under registration at https://www.humanconnectome.org/. Specific code for the analyses, patient’s lesion masks normalized to common template, lesion volumes, dis-connectivity structural and functional maps from stroke patients and their behavioral data are available under request to corresponding author.

## Abbreviations

ARAT: Action Research Arm Test
BOLD: Blood-oxygenation-level-dependent
CCA: Canonical correlation analysis
CST: Corticospinal tract
Em-NSA: Erasmus-modified Nottingham Sensory Assessment
DBS: Deep brain stimulation
EPI: Echo-planar imaging
FC: Functional connectivity
FD: Frame-wise displacements
FLAIR: Fluid-attenuated inversion recovery
FMA-UE: Fugl-Meyer assessment—upper extremity
FOV: Field of view
FWHM: Full width at half maximum
HCP: Human Connectome Project
LSM: Lesion symptom mapping
LNM: Lesion network mapping
LOOCV: Leave-one-out cross-validation
M1: Primary motor area
MCP: Middle cerebellar peduncle
MPRAGE: Magnetization prepared-rapid gradient echo
PCA: Principal component analysis
PTT: Perceptual threshold of touch
RSN: Resting state networks
SBC: Seed-based connectivity
SC: Structural connectivity
T: Maximum significant correlation
TBSAA: Time between stroke and assessment
TE: Echo time
TENS: Transcutaneous electrical nerve stimulation
TR: Repetition time
VAN: Ventral attention network
Ve: Variance explained
