# Supplementary material for: Multimodal and multidomain lesion network mapping enhances prediction of sensorimotor behavior in stroke patients
Source: Sci Rep. 2022 Dec 27;12:22400. doi: 10.1038/s41598-022-26945-x (PMC9794717; doi:10.1038/s41598-022-26945-x)
Supplement: Supplementary file 1 — Supplementary Information. [file 41598_2022_26945_MOESM1_ESM.docx]

**Supplementary material**

**Table S1. Anatomical description of lesion brain maps (resulting from Lesion Symptom Mapping) corresponding to the CCA solution and providing maximum association with sensorimotor behavior.** Labels correspond to the atlas Desikan-Killiany. Only shown regions with Z > 2 and percentage of overlap > 50 voxels.

| Brain Area | Peak x | Peak y | Peak z | Zmax | Nvoxels | Perc. Overlap |
| --- | --- | --- | --- | --- | --- | --- |
| ctx-rh-supramarginal | 32 | -26 | 26 | 7.17 | 999 | 39.30 |
| ctx-rh-insula | 30 | -6 | 18 | 8.45 | 650 | 28.08 |
| rh-Thalamus-proper | 18 | -12 | 16 | 3.96 | 605 | 49.35 |
| ctx-rh-postcentral | 42 | -16 | 28 | 6.87 | 555 | 24.25 |
| ctx-rh-precentral | 46 | 0 | 30 | 6.51 | 467 | 10.90 |
| ctx-lh-supramarginal | -36 | -28 | 22 | 4.80 | 438 | 13.70 |
| lh-Thalamus-Proper | -18 | -24 | 18 | 5.80 | 301 | 24.55 |
| ctx-lh-insula | -28 | -34 | 20 | 6.77 | 226 | 9.97 |
| rh-Putamen | 28 | -6 | 6 | 8.07 | 205 | 24.29 |
| ctx-rh-superiortemporal | 58 | -28 | 16 | 5.44 | 140 | 4.87 |
| lh-Putamen | -20 | 6 | 6 | 4.11 | 137 | 14.76 |
| ctx-lh-superiortemporal | -42 | -40 | 20 | 3.38 | 83 | 2.75 |
| ctx-lh-inferiorparietal | -44 | -50 | 22 | 3.32 | 74 | 2.12 |
| ctx-rh-transversetemporal | 36 | -24 | 10 | 3.47 | 73 | 33.80 |
| lh-Caudate | -20 | -28 | 18 | 6.40 | 71 | 11.04 |

**Table S2. Anatomical description of lesion maps (resulting from Lesion Symptom Mapping) corresponding to the CCA solution and providing maximum association with sensorimotor behavior.** Labels correspond to the atlas XTRACT. Only shown tracts with Z > 2 and percentage of overlap > 50 voxels.

| Tract | Peak x | Peak y | Peak z | | Zmax | Nvoxels | Perc. Overlap |
| --- | --- | --- | --- | --- | --- | --- | --- |
| Arcuate Fasciculus R | 34 | -2 | 18 | 7.76 | | 493 | 32.31 |
| Superior Thalamic Radiation R | 28 | -12 | 30 | 6.95 | | 442 | 22.831 |
| Corticospinal Tract R | 24 | -22 | 22 | 10.17 | | 386 | 17.58 |
| Arcuate Fasciculus L | -30 | -30 | 24 | 5.73 | | 292 | 20.78 |
| Superior Longitudinal Fasciculus 3 L | -48 | -42 | 32 | 3.57 | | 201 | 9.43 |
| Superior Thalamic Radiation L | -18 | -24 | 16 | 6.40 | | 188 | 9.52 |
| Corticospinal Tract L | -22 | -26 | 16 | 6.40 | | 184 | 8.63 |
| Superior Longitudinal Fasciculus 2 R | 30 | -2 | 38 | 5.68 | | 173 | 6.68 |
| Middle Longitudinal Fasciculus L | -26 | -36 | 18 | 6.19 | | 156 | 11.49 |
| Superior Longitudinal Fasciculus 3 R | 40 | -16 | 28 | 7.89 | | 120 | 6.04 |
| Acoustic Radiation L | -26 | -34 | 18 | 6.09 | | 87 | 12.61 |
| Inferior Fronto-Occipital Fasciculus R | 32 | -8 | -4 | 4.46 | | 73 | 4.18 |
| Inferior Fronto-Occipital Fasciculus L | -24 | 4 | -12 | 3.91 | | 69 | 4.37 |
| Superior Longitudinal Fasciculus 2 L | -30 | -46 | 28 | 3.32 | | 66 | 2.41 |
| Optic Radiation L | -28 | -36 | 18 | 5.64 | | 60 | 6.34 |
| Acoustic Radiation R | 30 | -26 | 4 | 3.37 | | 58 | 8.964 |
| Middle Longitudinal Fasciculus R | 30 | -22 | 10 | 7.63 | | 54 | 3.954 |
| Anterior Thalamic Radiation L | -18 | -8 | 20 | 4.11 | | 50 | 2.74 |

**Table S3. Anatomical description of unimodal SC Lesion Network Mapping brain maps corresponding to the CCA solution and providing maximum association with sensorimotor behavior.** Labels correspond to the atlas XTRACT. Only shown tracts with Z > 2 and percentage of overlap > 50 voxels.

| Tract | Peak x | Peak y | Peak z | Zmax | Nvoxels | Perc. Overlap |
| --- | --- | --- | --- | --- | --- | --- |
| Forceps Major | -8 | -34 | 12 | 4.66 | 1157 | 39.54 |
| Frontal Aslant Tract L | -16 | 8 | 30 | 4.76 | 890 | 60.26 |
| Superior Thalamic Radiation L | -16 | 8 | 44 | 4.41 | 875 | 44.30 |
| Superior Longitudinal Fasciculus 1 L | -16 | -4 | 42 | 4.67 | 731 | 31.56 |
| Optic Radiation L | -28 | -30 | 4 | 4.20 | 668 | 71.14 |
| Superior Longitudinal Fasciculus 2 L | -28 | 24 | 26 | 3.37 | 645 | 23.59 |
| Inferior Fronto-Occipital Fasciculus L | -26 | -52 | 20 | 4.63 | 482 | 30.51 |
| Middle Longitudinal Fasciculus L | -28 | -58 | 20 | 4.53 | 452 | 33.28 |
| Anterior Thalamic Radiation L | -18 | 32 | 22 | 3.87 | 414 | 22.66 |
| Fornix L | -10 | -34 | 12 | 4.50 | 411 | 40.69 |
| Forceps Minor | -12 | 22 | 16 | 5.48 | 358 | 19.47 |
| Cingulum subsection: Dorsal L | -10 | 0 | 30 | 4.60 | 314 | 22.49 |
| Acoustic Radiation L | -26 | -28 | 4 | 3.91 | 214 | 31.01 |
| Frontal Aslant Tract R | 16 | 14 | 26 | 4.26 | 212 | 13.55 |
| Corticospinal Tract L | -12 | -20 | 58 | 3.19 | 192 | 9.01 |
| Superior Longitudinal Fasciculus 1 R | 12 | 6 | 24 | 4.02 | 188 | 7.67 |
| Superior Thalamic Radiation R | 18 | -2 | 38 | 3.43 | 139 | 7.18 |
| Cingulum subsection: Temporal L | -24 | -42 | -2 | 2.82 | 137 | 16.47 |
| Vertical Occipital Fasciculus L | -18 | -82 | 18 | 2.96 | 126 | 8.36 |
| Anterior Thalamic Radiation R | 20 | 30 | 22 | 3.75 | 96 | 5.08 |
| Uncinate Fasciculus L | -36 | -6 | -20 | 2.96 | 93 | 8.32 |
| Anterior Commissure | -28 | -8 | -10 | 3.55 | 88 | 28.12 |
| Arcuate Fasciculus L | -32 | -34 | 18 | 2.80 | 75 | 5.34 |
| Inferior Longitudinal Fasciculus L | -40 | -16 | -20 | 2.89 | 73 | 3.47 |
| Optic Radiation R | 24 | -44 | 24 | 3.19 | 52 | 6.25 |

**Table S4. Anatomical description of multimodal SC Lesion Network Mapping brain maps corresponding to the CCA solution and providing maximum association with sensorimotor behavior.** Labels correspond to the atlas XTRACT. Only shown tracts with Z > 2 and percentage of overlap > 50 voxels.

| Tract | Peak x | Peak y | Peak z | Zmax | Nvoxels | Perc. Overlap |
| --- | --- | --- | --- | --- | --- | --- |
| Forceps Major | -8 | -34 | 12 | 4.68 | 1720 | 58.78 |
| Corticospinal Tract R | 8 | -22 | -26 | 5.46 | 779 | 35.47 |
| Anterior Thalamic Radiation L | -6 | -16 | 8 | 3.69 | 560 | 30.65 |
| Middle Cerebellar Peduncle | 2 | -20 | -32 | 4.16 | 541 | 19.77 |
| Frontal Aslant Tract L | -14 | 22 | 38 | 3.03 | 485 | 32.84 |
| Optic Radiation L | -24 | -76 | 14 | 4.27 | 467 | 49.73 |
| Forceps Minor | -10 | 24 | 12 | 4.61 | 434 | 23.60 |
| Optic Radiation R | 24 | -50 | 30 | 4.39 | 357 | 42.91 |
| Superior Longitudinal Fasciculus 1 L | -22 | -56 | 34 | 3.11 | 345 | 14.90 |
| Middle Longitudinal Fasciculus R | 26 | -50 | 28 | 4.56 | 320 | 23.43 |
| Middle Longitudinal Fasciculus L | -24 | -74 | 16 | 4.27 | 264 | 19.44 |
| Fornix L | -10 | -34 | 12 | 4.55 | 253 | 25.05 |
| Superior Longitudinal Fasciculus 1 R | 24 | -58 | 32 | 3.87 | 242 | 9.88 |
| Cingulum subsection: Dorsal L | -12 | 28 | 16 | 3.98 | 216 | 15.47 |
| Anterior Commissure | 28 | -12 | -4 | 4.98 | 190 | 60.70 |
| Inferior Fronto-Occipital Fasciculus R | 30 | -18 | 2 | 5.34 | 181 | 10.36 |
| Inferior Fronto-Occipital Fasciculus L | -26 | -52 | 18 | 4.70 | 173 | 10.95 |
| Vertical Occipital Fasciculus L | -18 | -82 | 18 | 3.85 | 154 | 10.21 |
| Superior Thalamic Radiation R | 22 | -18 | 44 | 3.78 | 145 | 7.49 |
| Acoustic Radiation R | 32 | -28 | 8 | 4.12 | 108 | 16.69 |
| Corticospinal Tract L | -26 | -16 | -2 | 4.22 | 98 | 4.60 |
| Superior Longitudinal Fasciculus 3 R | 38 | -2 | 28 | 2.78 | 78 | 3.92 |
| Acoustic Radiation L | -28 | -24 | 4 | 4.82 | 77 | 11.16 |
| Superior Longitudinal Fasciculus 2 R | 36 | -4 | 32 | 2.89 | 65 | 2.51 |
| Superior Longitudinal Fasciculus 2 L | -30 | -44 | 28 | 2.33 | 63 | 2.30 |

**Table S5. Percentage of overlapping between the unimodal SC Lesion Network Mapping brain maps and major sensorimotor brain tracts.** Labels correspond to the atlas SMATT. Only shown tracts with Z > 2 and percentage of overlap > 10 voxels. Abbreviations: M1 = primary motor cortex; PMd = dorsal premotor cortex; PMv = ventral premotor cortex; preSMA = pre-supplementary motor area; S1 = primary somatosensory cortex; SMA = supplementary motor area.

| Tract | Peak x | Peak y | Peak z | Zmax | Nvoxels | Perc. Overlap |
| --- | --- | --- | --- | --- | --- | --- |
| Left-PMd | -14 | -6 | 56 | 3.88 | 201 | 72.30 |
| Left-PMv | -14 | 10 | 50 | 4.17 | 196 | 70.76 |
| Right-M1,PMd,PMv,SMA,preSMA,S1 | -18 | -10 | 56 | 3.53 | 167 | 66.53 |
| Right-M1,PMd,PMv,SMA,preSMA | -30 | -18 | 62 | 3.01 | 116 | 19.66 |
| Right-preSMA | 18 | 0 | 42 | 3.18 | 52 | 18.51 |
| Right-S1 | 44 | -24 | 48 | 2.48 | 52 | 17.93 |
| Left-M1,SMA | -16 | -4 | 58 | 3.53 | 52 | 61.91 |
| Right-SMA | 18 | -6 | 44 | 3.28 | 51 | 17.59 |
| Left-PMd,SMA | -14 | 0 | 58 | 3.63 | 39 | 79.59 |
| Left-M1 | -22 | -4 | 30 | 2.64 | 25 | 7.31 |
| Right-PMd | 20 | -10 | 46 | 2.44 | 11 | 4.35 |
| Right-SMA,preSMA | 18 | -2 | 44 | 3.25 | 11 | 28.95 |
| Left-PMd,PMv | -20 | 2 | 44 | 3.17 | 11 | 64.71 |
| Left-M1,PMd,SMA,S1 | -18 | -4 | 52 | 3.38 | 11 | 22.45 |

**Table S6. Percentage of overlapping between the multimodal SC Lesion Network Mapping brain maps and major sensorimotor brain tracts.** Labels correspond to the atlas SMATT. Only shown tracts with Z > 2 and percentage of overlap > 10 voxels. Circuits with less than 10 voxels but with a high overlap (> 50%) are also shown. Abbreviations: M1 = primary motor cortex; PMd = dorsal premotor cortex; PMv = ventral premotor cortex; preSMA = pre-supplementary motor area; S1 = primary somatosensory cortex; SMA = supplementary motor area.

| Tract | Peak x | Peak y | Peak z | Zmax | Nvoxels | Perc. Overlap |
| --- | --- | --- | --- | --- | --- | --- |
| Right-M1 | 22 | -24 | 46 | 4.27 | 166 | 27.90 |
| Right-M1,S1 | 10 | -22 | -26 | 5.07 | 156 | 43.33 |
| Right-M1,PMd,PMv,SMA | 4 | -20 | -28 | 4.87 | 32 | 55.17 |
| Left-PMv | -6 | -20 | -24 | 3.58 | 31 | 11.19 |
| Right-M1,PMd,SMA,preSMA,S1 | 6 | -22 | -28 | 5.43 | 28 | 100.00 |
| Right-M1,PMv,S1 | 10 | -20 | -20 | 5.12 | 21 | 100.00 |
| Right-PMd | 22 | -16 | 46 | 3.53 | 19 | 7.51 |
| Right-PMv | 20 | -14 | 0 | 3.68 | 16 | 4.56 |
| Right-PMd,PMv,SMA,preSMA | 4 | -22 | -30 | 5.44 | 16 | 100.00 |
| Right-preSMA | 8 | -16 | -18 | 4.63 | 15 | 5.34 |
| Right-PMd,PMv,SMA | 8 | -22 | -26 | 5.46 | 15 | 100.00 |
| Right-M1,PMd,PMv,SMA,S1 | 8 | -16 | -20 | 4.97 | 14 | 100.00 |
| Right-M1,SMA,S1 | 18 | -12 | -8 | 4.33 | 12 | 57.14 |
| Right-SMA | 14 | -16 | -12 | 4.34 | 11 | 3.79 |
| Right-PMd,SMA,preSMA | 8 | -20 | -26 | 5.19 | 11 | 100.00 |
| Left-M1,PMd,SMA,preSMA | -4 | -24 | -32 | 3.82 | 8 | 57.14 |
| Right-M1,SMA | 16 | -16 | -12 | 4.35 | 7 | 100.00 |
| Right-M1,PMv,SMA | 20 | -14 | -2 | 3.99 | 5 | 83.33 |
| Right-M1,PMv,SMA,S1 | 14 | -16 | -14 | 4.49 | 4 | 100.00 |
| Right-M1,PMd,SMA | 18 | -12 | -10 | 4.69 | 2 | 100.00 |
| Right-M1,PMd,SMA,preSMA | 6 | -24 | -28 | 5.00 | 2 | 100.00 |
| Right-M1,PMd,SMA,S1 | 4 | -28 | -34 | 4.05 | 1 | 100.00 |

**Table S7. Anatomical description of unimodal FC Lesion Network Mapping brain maps corresponding to the CCA solution and providing maximum association with sensorimotor behavior.** Labels correspond to the atlas Desikan-Killiany. Only shown regions with Z > 2 and percentage of overlap > 50 voxels.

| Brain Area | Peak x | Peak y | Peak z | Zmax | Nvoxels | Perc. Overlap |
| --- | --- | --- | --- | --- | --- | --- |
| Brain stem | 4 | -24 | -32 | 8.02 | 1287 | 35.63 |
| ctx-rh-superiorfrontal | 10 | 10 | 66 | 5.07 | 716 | 11.11 |
| rh-Thalamus-proper | 18 | -16 | 4 | 3.82 | 715 | 58.32 |
| ctx-lh-supramarginal | -58 | -28 | 24 | 4.08 | 616 | 19.27 |
| lh-Thalamus-Proper | -16 | -14 | 10 | 6.36 | 554 | 45.19 |
| ctx-lh-superiorfrontal | -14 | 14 | 64 | 3.45 | 329 | 5.11 |
| ctx-lh-inferiorparietal | -54 | -60 | 16 | 3.43 | 265 | 7.58 |
| ctx-lh-middletemporal | -56 | -60 | 14 | 3.44 | 261 | 10.44 |
| ctx-lh-insula | -28 | -18 | 10 | 3.46 | 228 | 10.06 |
| ctx-rh-precentral | 42 | -8 | 34 | 3.01 | 211 | 4.93 |
| ctx-lh-precuneus | -6 | -74 | 38 | 2.40 | 134 | 4.57 |
| lh-Putamen | -28 | -18 | 6 | 3.69 | 126 | 13.58 |
| ctx-lh-bankssts | -52 | -56 | 16 | 3.22 | 118 | 12.43 |
| ctx-rh-postcentral | 58 | -6 | 30 | 2.48 | 84 | 3.67 |
| ctx-rh-supramarginal | 54 | -26 | 26 | 2.86 | 78 | 3.07 |
| ctx-lh-inferiortemporal | -54 | -58 | 4 | 2.83 | 77 | 2.36 |

**Table S8. Anatomical description of multimodal FC Lesion Network Mapping brain maps corresponding to the CCA solution and providing maximum association with sensorimotor behavior.** Labels correspond to the atlas Desikan-Killiany. Only shown regions with Z > 2 and percentage of overlap > 50 voxels.

| Brain Area | Peak x | Peak y | Peak z | Zmax | Nvoxels | Perc. Overlap |
| --- | --- | --- | --- | --- | --- | --- |
| Brain stem | 2 | -24 | -42 | 19.76 | 917 | 25.39 |
| lh-Thalamus-Proper | -18 | -16 | 10 | 8.19 | 580 | 47.31 |
| ctx-lh-supramarginal | -58 | -30 | 24 | 3.99 | 529 | 16.55 |
| ctx-lh-inferiorparietal | -42 | -84 | 26 | 3.28 | 444 | 12.70 |
| ctx-rh-superiorfrontal | 14 | 18 | 64 | 3.17 | 370 | 5.74 |
| ctx-rh-precentral | 40 | -10 | 34 | 5.93 | 283 | 6.61 |
| ctx-lh-precuneus | -16 | -62 | 24 | 2.39 | 167 | 5.69 |
| ctx-lh-middletemporal | -54 | -66 | 12 | 2.76 | 165 | 6.60 |
| ctx-lh-superiorfrontal | -10 | 12 | 66 | 2.87 | 149 | 2.31 |
| ctx-lh-inferiortemporal | -52 | -62 | 4 | 2.74 | 118 | 3.62 |
| ctx-rh-postcentral | 44 | -12 | 32 | 4.47 | 115 | 5.02 |
| ctx-lh-insula | -28 | -20 | 8 | 2.58 | 104 | 4.59 |
| ctx-rh-precuneus | 18 | -62 | 26 | 2.50 | 100 | 3.13 |
| ctx-lh-lateraloccipital | -48 | -76 | 20 | 2.60 | 91 | 2.25 |
| lh-Caudate | -18 | -16 | 20 | 4.74 | 63 | 9.80 |
| ctx-lh-fusiform | -30 | -44 | -10 | 2.99 | 59 | 2.40 |
| lh-Putamen | -28 | -20 | 4 | 2.66 | 52 | 5.60 |

**Table S9. Percentage of overlapping between the unimodal FC Lesion Network Mapping brain maps and major resting state networks.** Labels correspond to the Yeo's partition.

| RSN | Peak x | Peak y | Peak z | Zmax | Nvoxels | Perc. Overlap |
| --- | --- | --- | --- | --- | --- | --- |
| Dorsal Attention | -18 | -14 | 10 | 6.57 | 2214 | 10.57 |
| Limbic | 8 | 12 | 66 | 4.80 | 990 | 2.59 |
| Frontoparietal | -14 | -12 | 14 | 4.82 | 867 | 3.09 |
| Sensory-motor | -18 | -22 | 8 | 5.24 | 824 | 3.43 |
| DMN | -18 | -26 | 8 | 3.72 | 535 | 3.07 |
| Ventral Attention | -20 | -26 | 8 | 3.85 | 278 | 1.25 |
| Visual | 10 | -28 | -38 | 5.96 | 138 | 0.85 |

**Table S10. Percentage of overlapping between the multimodal FC Lesion Network Mapping brain maps and major resting state networks.** Labels correspond to the Yeo's partition

| RSN | Peak x | Peak y | Peak z | Zmax | Nvoxels | Perc. Overlap |
| --- | --- | --- | --- | --- | --- | --- |
| Ventral Attention | -18 | -16 | 12 | 8.4399 | 1172 | 5.5967 |
| Sensory-motor | -18 | -22 | 8 | 6.7411 | 705 | 2.9359 |
| DMN | -14 | -30 | 10 | 3.2307 | 687 | 1.7943 |
| Dorsal Attention | -18 | -26 | 10 | 5.7506 | 652 | 3.7402 |
| Visual | -22 | -26 | 8 | 6.0141 | 557 | 2.504 |
| Frontoparietal | -14 | -12 | 14 | 5.5747 | 389 | 1.3865 |
| Limbic | 10 | -28 | -36 | 7.3685 | 33 | 0.20245 |

**
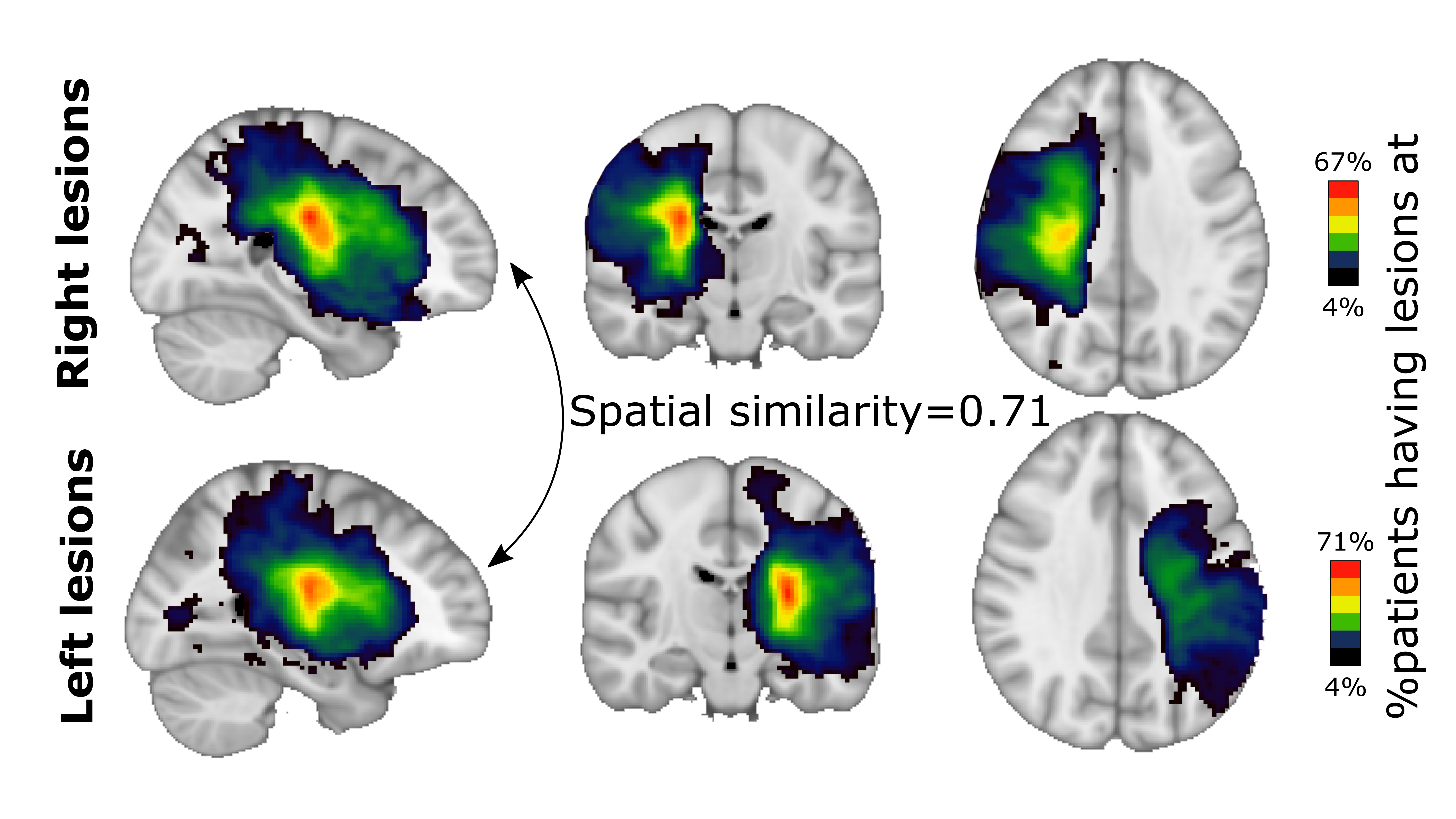
**

**Figure S1. Comparison between lesion maps at right vs left hemispheres.** Top panel: Among patients who have the stroke lesion in the right hemisphere, brain maps show the percentage of patients within this group who share the same location within the lesion. Lower panel: The same but for the group of patients who have the lesion in the left hemisphere. The spatial similarity between the two maps was 0.71, indicating a large amount of overlap between them, and that by looking at location maps the two groups of patients are difficult to differentiate (those with the lesion in the right hemisphere compared to those with the lesion in the left).


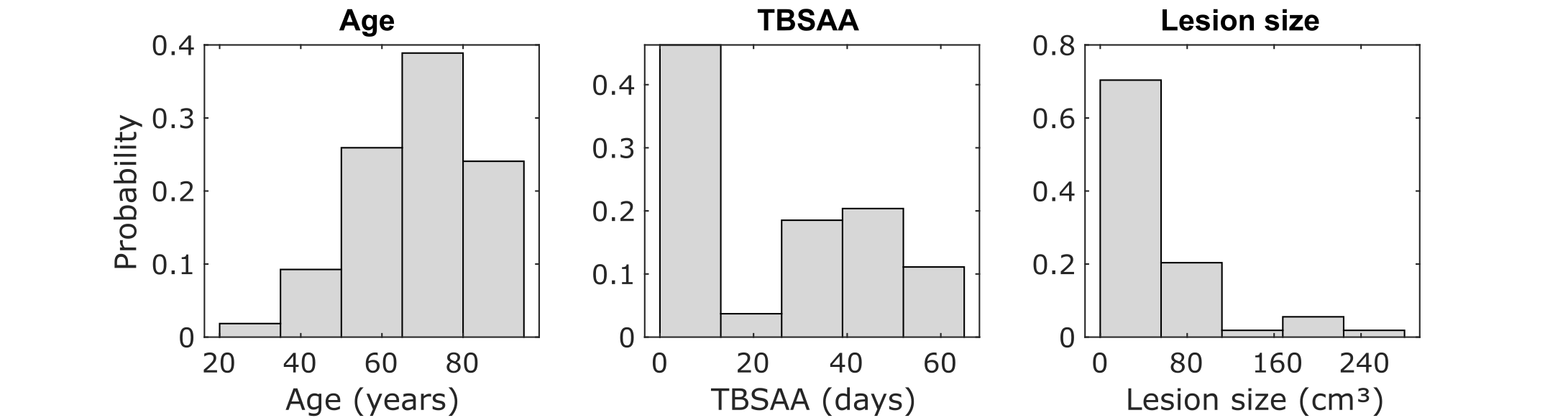


**Figure S2. Probability distributions of covariates regressed out from sensorimotor variables.**


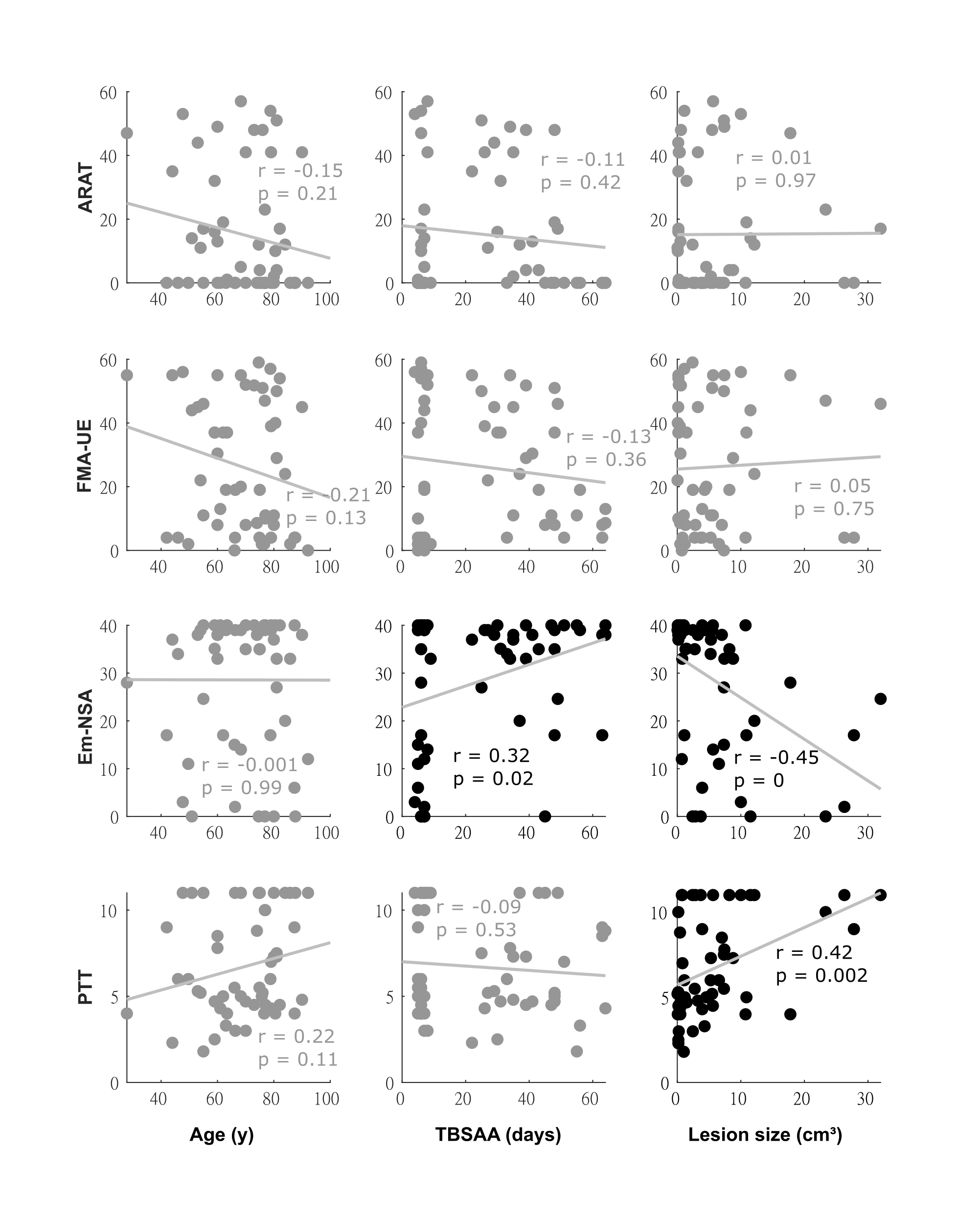


**Figure S3. Relation between behavioral -sensorimotor- scores and covariables age, days between stroke and assessment, and lesion size).** The two first rows correspond to motor scores (ARAT, FMA-UE), and the third and fourth to sensory scores (Em-NAS, PTT). Most of scores show a statistical tendency to correlate with the cofounders (that has been regress-out in our analyses). Scatter plots with significant correlation values (p<0.05) have been highlighted in black. Abbreviations TBSAA = time between stroke and assessment.


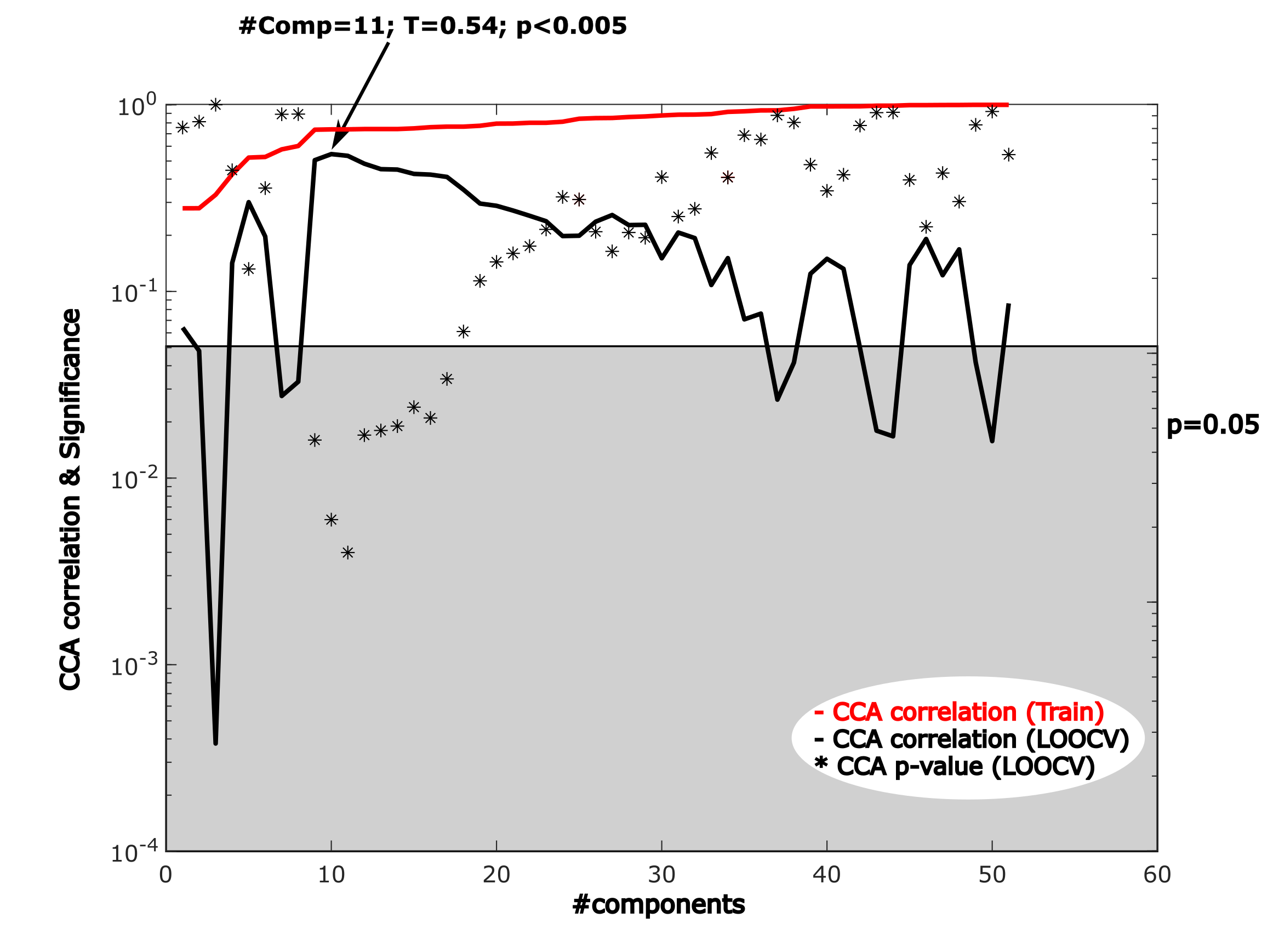


**Figure S4. Methodological sketch for CCA analysis for maximum behavioral association across imaging modalities.** For each imaging modality (structural or functional) we obtained matrices of size Npatients $\times$Nvoxels, and applied PCA to obtain structural and functional components equal to the number of patients. With those components used as independent variables (input), we applied CCA using the behavioral scores as dependent variables (output). The train curve, here represented in red, shows that when the number of components increases, the input-output correlations monotonously increase, indicating over-fitting. Here, we applied leave-on-out cross-validation (LOOCV) to overcome this problem, represented here with the black curve. Maximum correlations values (represented by T) are calculated, and also reported their corresponding p-values as a measure for statistical significance. At this point of maximum correlation, the brain maps corresponding to each modality are obtained (which are shown in Figures 3 and 4). Here, a log-linear representation of the data has been chosen.





**Figure S5. Brain maps with maximum behavioral-association for single modality (LSM and LNM) and multi-modal LNM after applying CCA and no thresholding the maps.** Similar to Figures 3 and 4 but without perform a z-score and neither thresholding the maps. PCA+CCA weights were normalized to be between 0 and 1.


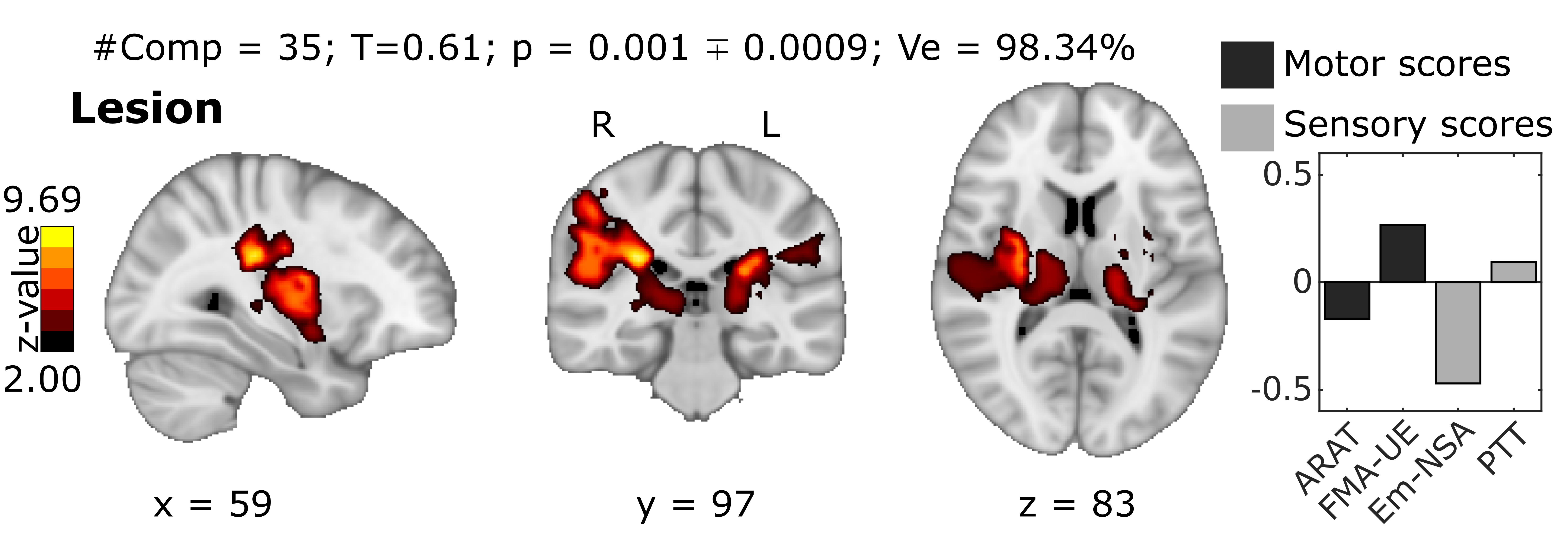


**Figure S6. Brain maps with maximum behavioral-association for the lesion modality without removal of the effect of lesion size.** Similar to Figure 3, but without eliminating the effect of lesion size. Notice that, when the lesion size effect is not eliminated, the T value is considerably higher as compared to the case of not eliminating it (T=0.61 vs T=0.38).

**

**

**Figure S7. Brain maps with maximum behavioral-association for single modality and multi-modal CCA without elimination of the effect of lesion size.** Similar to Figure 4, in which the covariable lesion size was regressed-out in the behavioral scores, but without regressing it. Major variations with respect to Figure 4 correspond to increased performance achieved by SC, and different behavioral weights represented in the right panels in each row.
